# Genotyping of whole genome amplified reduced representation libraries reveals a cryptic population of *Culicoides brevitarsis* in the Northern Territory, Australia

**DOI:** 10.1186/s12864-016-3124-1

**Published:** 2016-09-30

**Authors:** Maria G. Onyango, Nicola C. Aitken, Cameron Jack, Aaron Chuah, James Oguya, Appolinaire Djikeng, Steve Kemp, Glenn A. Bellis, Adrian Nicholas, Peter J. Walker, Jean-Bernard Duchemin

**Affiliations:** 1 CSIRO Health and Biosecurity, Australian Animal Health Laboratory, 5 Portalington Road, Geelong, 3220 VIC Australia; 2 School of Medicine, Deakin University, 75 Pidgons Road, Waurn Ponds, 3216 VIC Australia; 3 Research School of Biology, Australian National University, Canberra, ACT 2601 Australia; 4 The John Curtin School of Medical Research, Australian National University, Canberra, ACT 2601 Australia; 5 International Livestock Research Institute (ILRI), P.O. Box 30709, 00100 Nairobi, Kenya; 6 Biosciences eastern and central Africa—ILRI Hub (BecA-ILRI Hub), ILRI, PO Box 30709, 00100 Nairobi, Kenya; 7 Northern Australia Quarantine Strategy, 1 Pederson Road, Marrara, 0812 NT Australia; 8 Research Institute for the Environment and Livelihoods, Charles Darwin University, Darwin, 0909 NT Australia; 9 NSW Department of Primary Industries, Biosecurity, 4 Marsden Park Road, Calala, 2340 NSW Australia

**Keywords:** *Culicoides brevitarsis*, GBS, SNPs, Bluetongue virus, Australia

## Abstract

**Background:**

The advent of genotyping by Next Generation Sequencing has enabled rapid discovery of thousands of single nucleotide polymorphism (SNP) markers and high throughput genotyping of large populations at an affordable cost. Genotyping by sequencing (GBS), a reduced representation library sequencing method, allows highly multiplexed sequencing of genomic subsets. This method has limitations for small organisms with low amounts of genomic DNA, such as the bluetongue virus (BTV) vectors, *Culicoides* midges.

**Results:**

This study employed the GBS method to isolate SNP markers *de novo* from whole genome amplified *Culicoides brevitarsis* genomic DNA. The individuals were collected from regions representing two different Australian patterns of BTV strain distribution: the Northern Territory (NT) and the east coast.

We isolated 8145 SNPs using GBS. Phylogenetic analysis conducted using the filtered 3263 SNPs revealed the presence of a distinct *C. brevitarsis* sub-population in the NT and this was confirmed by analysis of mitochondrial DNA. Two loci showed a very strong signal for selection and were unique to the NT population. Bayesian analysis with STRUCTURE indicated a possible two-population cluster.

**Conclusions:**

The results suggest that genotyping vectors with high density markers in combination with biological and environmental data is useful. However, more extensive sampling over a wider spatial and temporal range is needed. The presence of sub-structure in populations and loci under natural selection indicates the need for further investigation of the role of vectors in shaping the two Australian systems of BTV transmission. The described workflow is transferable to genotyping of small, non-model organisms, including arthropod vectors of pathogens of economic and medical importance.

**Electronic supplementary material:**

The online version of this article (doi:10.1186/s12864-016-3124-1) contains supplementary material, which is available to authorized users.

## Background

Advances in next generation sequencing technology have provided access to studies of whole genome variation. This technological jump has pushed the fields of population genetics and phylogeography to a higher level of marker density than was conceivable in the PCR era. Significant reduction of costs of whole genome sequencing [1] has led to a predictable shift of gold standard methods towards whole genome sequencing approaches, either whole genome resequencing or *de novo* DNA sequencing. This trend has already been applied to research on arthropod vectors in which knowledge of population structure, dispersal and gene flow, especially in the framework of vector control, is of paramount importance. To date, most studies using whole genome sequencing methods have targeted mosquitoes which serve as malaria [2] or arbovirus [3] vectors. In contrast, despite their role as vectors for arboviruses of veterinary and economical importance, and being widely spread in much of the world [4], research on *Culicoides* has not yet benefited from this technological jump. *Culicoides* genome size is about 200 Mb, similar to *Anopheles* mosquitoes [5].

However, as biting midges are very small in size (1–3 mm adult length) and only few species have been reared in the laboratory, genomics resources are rare for *Culicoides* species.

**Fig. 1:**
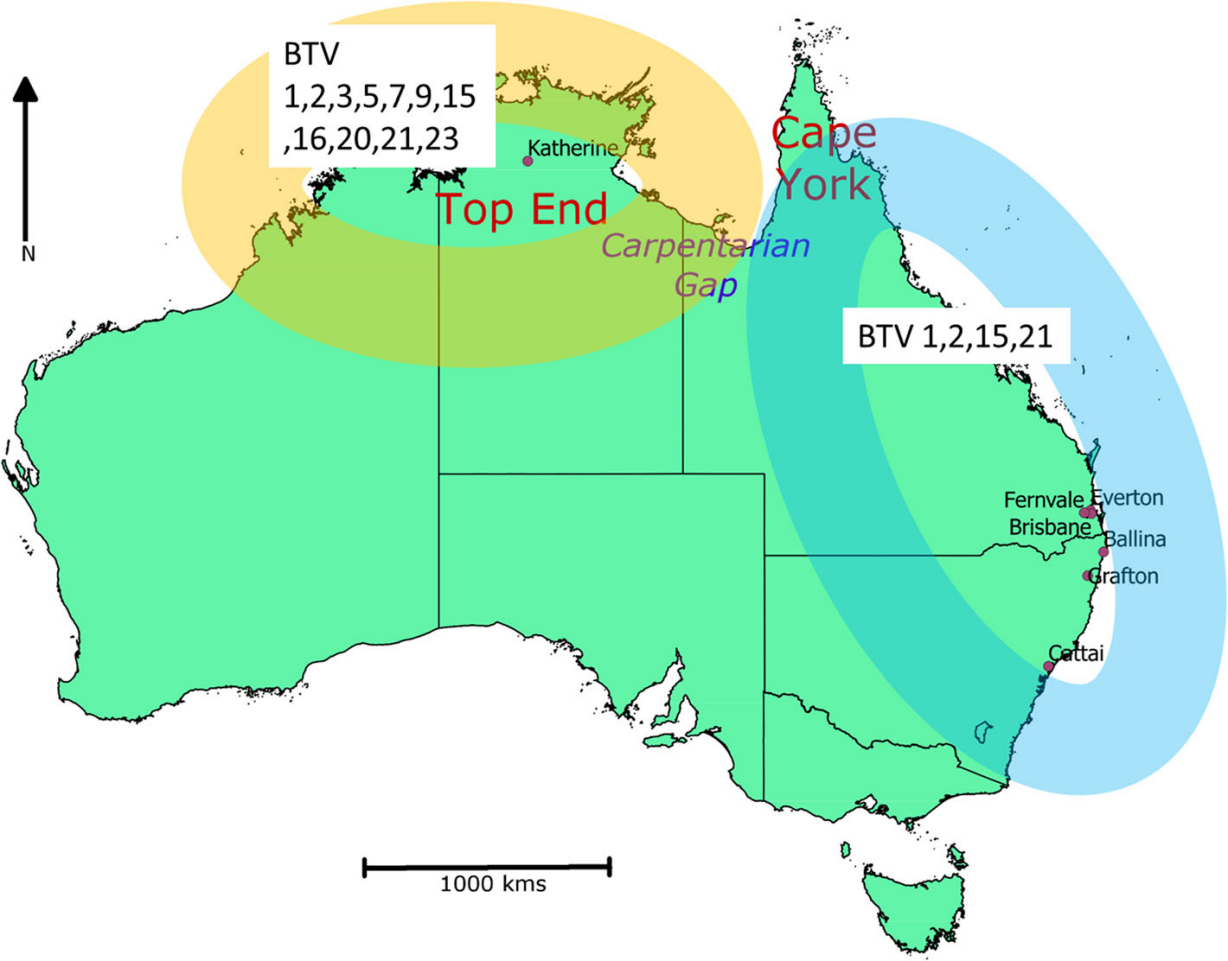
A map showing the collection sites and the distribution of BTV serotypes in Australia. The distribution of the BTV serotypes depicts the two BTV epidemiological systems: one in the north of Northern Territory (pale orange) and the other in the eastern states of Queensland and New South Wales (pale blue). The administrative limits of the Australian states and territories are marked with a continuous black line. The BTV serotypes are tagged with their respective distribution zones. The sites of collection of *Culicoides* are indicated as purple dots. Note the Carpentarian Gap (in blue) located between the two tropical zones of Cape York (northern Queensland) and the Top End (northern NT), at the junction of the BTV serotype distribution zones

*Culicoides brevitarsis* was first described in Australia in 1917 [6] and it is known to have a wide geographical distribution across the Oriental and Australasian regions [7]. In Australia, *C. brevitarsis* appears to be the principal vector of both bluetongue virus (BTV) and Akabane virus which cause economically significant infections of livestock [8, 9]. Since the initial detection of BTV in Australia in 1977, 12 serotypes (1, 2, 3, 5, 7, 9, 12, 15, 16, 20, 21, 23) have been isolated from the central northern region of Australia, in the Northern Territory (NT) [10, 11]. In contrast, only four serotypes (1, 2, 15 and 21) have been detected in the east of the continent with serotypes 1 and 21 being detected since the 1980s along the east coast down to the New South Wales, while serotypes 2 and 15 have been detected only since 2010, and only in Queensland (Qld) (Fig. 1) [10, 12, 13]. This suggests the existence of two Australian BTV systems of transmission (northern and eastern), including virus, vectors, potential hosts and environments, or episystems [14]. The reasons for these two distinct episystems in Australia are unknown.

The distribution of *C. brevitarsis* encompasses the northern part of Western Australia and the Northern Territory (NT), across the northern and east coastal regions of Queensland (Qld), to the narrow coastal strip of the northern half of New South Wales (NSW) [9]. Previous studies have shown that *C. brevitarsis* is able to migrate and maintain gene flow between distant populations, such as between Indonesia and Australia [15, 16]. Knowing if this vector species encounters barriers to its movement that could explain the BTV serotype distribution would be of great importance in understanding the risk of incursion of new serotypes into the eastern zone.

The Restriction Associated DNA sequencing (RADseq) technique, first described by Baird et al. in 2008 [17], is the most popular of reduced-representation library sequencing methods [18, 19]. It allows reduction of the complexity of genomes leading to deep sequence coverage of the fragments adjacent to the restriction site, subsequently leading to detection of SNPs [20]. It has several advantages over previous marker discovery tools such as restriction fragment length polymorphisms (RFLPs), amplified fragment length polymorphisms (AFLPs) and random amplified polymorphic DNA (RAPD) due to its ability to identify, verify and score markers concurrently. The RADseq technique is suitable for organisms without an existing reference genome [21]. It has been applied previously in phylogeography and population genetics of diverse organisms [19], including mosquitoes [2, 3, 22]. Among the techniques for detecting DNA sequence variation for conservation applications, and by extension to population genetics and phylogeography, RADSeq is one of the more economical [20]. Briefly, genomic DNA from several samples of interest is digested using a restriction enzyme of choice. Adapters containing barcodes that can identify uniquely the specific samples and an overhang corresponding to the restriction enzyme cut site are ligated to the digested fragments. The ligated fragments are sheared and bands in the size range of 300–700 bp are selected. These are subsequently amplified using polymerase chain reaction (PCR) before sequencing on an Illumina platform [23–25]. While the significance of the RADseq technique in population genetics studies cannot be overstated, a number of methods with simpler and cheaper library preparation steps have been described.

Genotyping by sequencing (GBS), another reduced representation library method [1], allows highly multiplexed sequencing of genomic subsets. The reduction strategy is similar to RADSeq, with the restriction site length defining the degree of reduction [23], and the potential use of double restriction for a higher reduction [26]. In the GBS method, the genomic DNA, the restriction enzyme of choice and adapters are added to the same well. The ligated fragments are amplified by PCR and subsequently sequenced on an Illumina platform. Compared to RADSeq, the shearing and size selection steps are eliminated in the GBS procedure, reducing the amount of hands-on time [6]. This method has been successfully used to study diversity in several species: black cottonwood (*Populus trichocarpa*) [27], wheat [10], a moth, the European corn borer (*Ostrinia nubilalis*) [28] and the bumble bee (*Bombus bifarius*) [29].

The hypothesis to be tested in the present study was that deeper sequence coverage and isolation of SNPs from *C. brevitarsis* DNA might lead to a better understanding of the gene flow pattern of this important BTV vector in Australia. Resolving this may help in understanding the role played by the migration and population structure of this vector in sustaining the epidemiological pattern of serotypes across the continent. Both RADseq and GBS have a significant limitation for organisms with scarce amounts of genomic DNA, such as *Culicoides* [4]. Pooling of individual samples limits SNP discovery to those with very high allele frequency in the general population and rare alleles are lost [30]. The first objective of this study was to overcome the limitations posed by pooling of samples by using whole genome amplified (WGA) DNA of *C. brevitarsis* individuals to isolate SNP markers generated by GBS. The second objective was to apply these SNP markers for population genetic studies on *C. brevitarsis* collected from the two Australian BTV episystems. Because of its useful properties for taxonomic resolution and previous use for several *Culicoides* species [7, 31–33], sequence variation in the mitochondrial marker cytochrome oxidase subunit I (COI) was chosen for comparison with population structure detected using the GBS generated SNPs.

The present study describes a simple workflow that allows the isolation of genetic markers from samples of individuals with limited yields of genomic DNA and the further analysis of population genetics. This simplified workflow has great potential for application in ecology, evolution and conservation.

## Methods

### Insect sampling and DNA preparation

This study did not require any ethics approval because the midges were collected from animal sheds within farms and with the consent of the farm owners. Minimal contact with livestock animals took place without any disturbance.

A total of 96 midges was obtained from seven sites in Australia [NT (1 site), Qld (3 sites) and NSW (3 sites)] (Fig. 1) (Table 1). The midges were collected using green LED light traps [34] set 1 h prior to sunset and collected at approximately 0800 h the following morning. The collected samples were transported to the laboratory in 70 % ethanol. Specimens were identified to species level based on the wing pattern [7] observed using a binocular microscope. Species identification was verified using genetic methods as described in Bellis et al. [7, 32] to ensure isomorphic species of *Culicoides* were not included in analyses. Total genomic DNA was extracted from individual specimens using the DNeasy blood and tissue kit (Qiagen, Valencia, USA) according to the manufacturer’s protocol. The genomic DNA was quantitated using a Qubit fluorometer using Qubit dsDNA HS Assay Kit (Life Technologies, Invitrogen, California, USA).

**Table 1:** A summary of the sites of collections of *Culicoides*

Region	Site	Year	*n*	Latitude	Longitude
NT	Katherine	2012	40	−14.24411782	132.4565168
Queensland	Fernvale	2013	4	−27.4559047	152.6532798
	Brisbane	2013	2	−27.4710107	153.0234489
	Everton park	2013	2	−27.4071662	152.9910401
NSW	Cattai	2013	21	−33.5599283	150.9074577
	Ballina	2012	21	−28.8684827	153.560001
	Grafton	2013	3	−29.6911226	152.9331993

### Whole genome amplification of *C. brevitarsis*

To increase the genomic DNA yield from individual midges (<100 ng), multiple displacement amplification (MDA)-based WGA was conducted on each individual using the Repli-g ultrafast mini kit (Qiagen, Valencia, USA) according to the manufacturer’s protocol [15, 33]. The amount of DNA used for each midge was about 1 ng. The resulting DNA was quantitated using a Qubit fluorometer and a Qubit dsDNA BR Assay Kit (Life Technologies, Invitrogen, California, USA) and visualised on 1 % agarose gel at 7.40 V/cm.

### GBS library preparation

GBS libraries were constructed in 96-plex using custom adapters and barcodes. Approximately 500 ng of DNA generated by WGA was combined with 2.25 ng adapter that included a *PstI* restriction overhang. The methodology for library construction was essentially that of Elshire et al. [1], except pairwise barcoding was used to enable multiplexing. Briefly, the whole genome amplified DNA of each individual was digested with *PstI* (CTGCAG) for genome complexity reduction and ligated with one of 96 unique pairs of barcoded sequencing adaptors. The barcoded samples were then PCR amplified using MyTaq HS 2× Mix (Bioline) according to the manufacturer’s specifications. Samples were individually quantitated and pooled in an equimolar manner. Library amplicons, 250–600 bp in length, were extracted and sequenced on an Illumina HiSeq2000 using a 100 bp Paired End protocol at the Biomolecular Resource Facility at the Australian National University, Canberra ACT.

### Analysis of Illumina raw reads using the UNEAK GBS pipeline

The raw reads of the sequenced GBS libraries were analyzed using the Universal Network Enabled Analysis Kit (UNEAK) GBS pipeline which is designed for taxa without a reference genome and is part of the TASSEL 3.0 bioinformatics analysis package [35, 36]. In this method, a tag (haplotype) is considered to be a unique sequence representing a group of reads. The default parameters in the UNEAK pipeline were used.

Reads were retained if they contained a barcode, a cut site, and had no indeterminate bases within the first 64 bp after the barcode. From the raw reads, each sequence was trimmed to 64 bp in length. Reads were first merged into individual taxon tag count files and then merged into a ‘master’ tag to include all the tags from the same taxon, keeping tags with total reads count greater than or equal to 5 per sample. Globally, tag pairs that differed by a single nucleotide were retained as SNPs.

### Post-UNEAK pipeline analysis

Isolated SNPs were analysed using R script [37] that we developed in this study (Additional file 1). The script filtered the *Culicoides* individuals with more than or equal to 5 % of the total SNPs isolated (8145 SNPs) and the SNPs present in more than or equal to 10 % of the total individuals (96 individuals). Once the SNPs and the samples selected, the script was used to calculate a genetic distance matrix and obtain a hierarchical cluster dendrogram. To limit the ordering bias of SNPs and samples in the data matrix, Pvclust [38] was used for assessing the uncertainty in hierarchical cluster analysis. *P* values are calculated through multiscale bootstrap resampling, by randomly shuffling the samples and/or SNPs a number of times (here 1000 times). Pvclust-generated dendogram has two types of *p* values—AU (Approximately Unbiased) and BP (Bootstrap Probability) value. AU is computed by multiscale bootstrap resampling: this is obtained by looking at the changes of frequencies of fallen replicates along changing sample sizes, at each topography of the tree [39]. BP is computed by normal bootstrap resampling, with a constant sample size. In this study, the default setting of 1000 bootstraps was used.

To infer population structure from the SNP data, multilocus genetic distance estimates, with Fst [40, 41] were calculated between two population pairs corresponding to the BTV episystems (NT and East Coast) using GenePop [42] and Arlequin [41]. Permutation tests (100 replications) were used to determine the significance of the population structure estimates.

Deviation from the Hardy-Weinberg equilibrium (HWE) was estimated by using GenAlex v6.502 [43]. The observed number of heterozygotes and homozygotes for each locus in each population was tested against expected values using a chi-square test.

### Detection of loci under natural selection

To detect any loci under natural selection, BayeScan v2.1 [44] was applied. It defines two alternative models; one includes the effect of selection and the other excludes it. Bayesian inference utilises a likelihood function that results in a quantity called the posterior probability. The posterior probability of being under the effect of selection was estimated for each given locus using a reversible-jump Markov Chain Monte Carlo approach (MCMC) which simulates random processes. Its computational algorithm repeatedly samples randomly in order to obtain numerical results. This statistical method allows control against false positives. A q-value (the false discovery rate analogue of the *p*-value) of 0.1 is considered stringent. A Bayes factor of 32–100 corresponding to a posterior probability of 0.97–1.00 is considered strongly indicative of loci under natural selection.

### Inferring population structure using a Bayesian model

To infer the presence of population structure, assign individuals to populations and identify admixed individuals, a Bayesian model-based approach in STRUCTURE v2.3 [45] was utilised. STRUCTURE assigns individuals a probability to belong to either a population. Selecting the ‘Admixture model’ option, allows the possibility for individuals, if their genotype is considered admixed, to be assigned to more than one population. A parameter set consisting of standard values of 100,000 ‘burnin’ period and 100,000 MCMC Reps after burnin was used. Within STRUCTURE, ‘Burnin’ refers to the practice of discarding the initial portion of an MCMC run. To estimate the optimal number of populations (K) fitting the data, Genodive v2.0 [46] was used. Individuals were clustered using analysis of molecular variance (AMOVA), and checked against a range of 1–10 theoretical clusters (or populations). Clumpak [47] was used to collate all the results obtained from these Genodive iterations and import them in the STRUCTURE software.

### Amplification and sequencing of standard mitochondrial gene *cytochrome c oxidase* subunit *I* (COI)

As described previously [32], primers *Bc1 Culic Fm* and *JerR2m* were used to PCR amplify a 692 bp segment of the *COI* gene from 76 *C. brevitarsis* individuals with accession numbers [GenBank: KX247448-KX247523]. A total of four previously identified and published haplotypes was added with the following accession numbers: [GenBank: KJ162968, KJ162967, KJ162975 and KJ162957]. These sequences included one of *C. asiatica*, the most closely related species to *C. brevitarsis* [7]. The PCR amplicons were purified using QIAquick PCR purification kit (Qiagen) and 20 μl was sequenced using the Sanger sequencing method (Macrogen, Geumchun-gu, Seoul).

### Phylogenetic analysis of mitochondrial DNA *(mtDNA)*

*COI* gene sequences were manually edited using Bioedit v7.1.9 [48] and aligned using MUSCLE [49]. The haplotype network was constructed in PopART [50] using the TCS Java program, estimating genealogy by calculating probabilities of DNA pairwise differences (95 % connection limit). DnaSP v5 [51] was used to estimate haplotype diversity. Pair-wise F_ST_ value estimates of genetic distances, Tajima’s D and Fu’s Fs tests of neutrality for the COI data per population were performed using Arlequin v3.5 [41].

## Results

### SNP marker isolation

A total of 16,102,542 reads was obtained from the 96-plex *Pst*I library resulting in an average of 167,734 reads per sample. Following filtration, the final genotype matrix contained genotypes of 3263 SNPs across 75 samples (Additional file 2).

### Intra- and interpopulation genetic differences

Hardy-Weinberg equilibrium tests conducted for the filtered SNPs in the two populations (NT and east coast) indicated that they deviated significantly from the HWE in 15 % of cases (975 of 6526 of for the two populations: 6526 = 2 × 3263 SNPs) (*P* < 0.001) (see Additional file 3). The F_ST_ values were significantly (*P* < 0.05) low (F_ST_ = 0.01) between NT and the east coast populations suggesting that the populations are genetically panmictic.

### Phylogenetic relationship

**Fig. 2:**
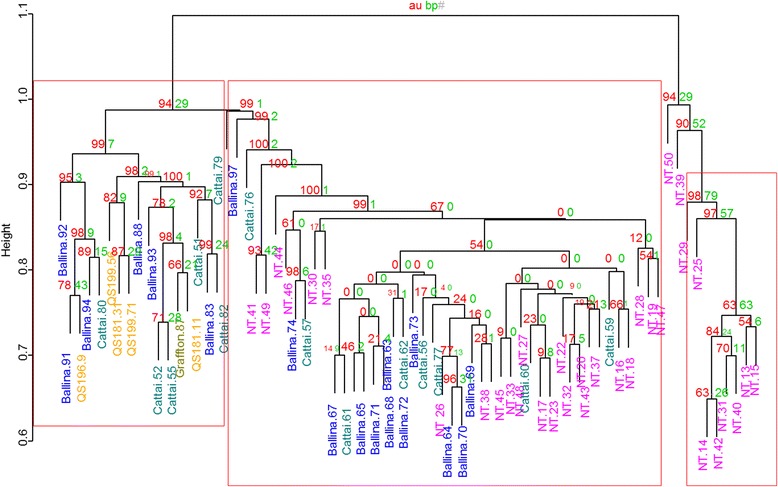
A dendogram showing the phylogenetic reconstruction based on filtered SNPs isolated from *C. brevitarsis* populations from two biogeographic regions of Australia. Numbers on inner branches represent *P* values calculated through multiscale bootstrap resampling. The red-colored *P* values represent the approximated unbiased (au) *P* values while the green *P* values represent the bootstrap probability (bp) *P* values (see main text for details). The red-framed clusters with AU *p*-value >/= 0.95 allow the rejection of an alternative hypothesis of non-existence of the clusters with a significance level of 0.05. The frame at the right contains only NT samples (pink); the left frame includes only East Coast samples (blue and green), with Queensland samples (yellow); and the central frame is constituted of mixed samples from the NT and the East Coast

The phylogenetic relationships of all populations were assessed using a dendrogram. A total of 10 individuals from NT samples clustered distinctly from all other individuals with high bootstrap support (>90 %). The remaining individuals from the NT clustered with the east coast individuals while all the few individuals from Qld clustered with the east coast samples (Fig. 2).

### Loci under natural selection

Two loci (TP 616 and TP 2560) showed very strong evidence for selection [P (α ≠ 0) = 0.97; q-value (0.03); log10 (PO) = 1.5] (Additional file 4). The two loci were detected among NT samples and were absent among east coast individuals. In the case of loci TP 616, 75 % of the individuals were found to be heterozygous while 30 % of the individuals were heterozygous at the TP 2560 locus.

### Population structure inference using a Bayesian modelling

**Fig. 3:**
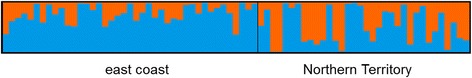
A plot of Bayesian clustering implemented in STRUCTURE for a probabilistic estimate of population structure. Individuals are represented by the vertical lines with K coloured segments representing the inferred clusters (K = 2) to which all the individuals are assigned probabilistically. The height of the bar corresponds to the probability of assignment to one theoretical population. The orange colour represents population 1 in the text, the blue as population 2

The best clustering model of population number of the individuals was K = 2 (Fig. 3). The proportion of membership of each pre-defined population (NT and east coast) in each of the two clusters was: East coast (population 1 = 0.139; population 2 = 0.861) and NT (population 1 = 0.492; population 2 = 0.508).

### Mitochondrial DNA haplotypes

**Fig. 4:**
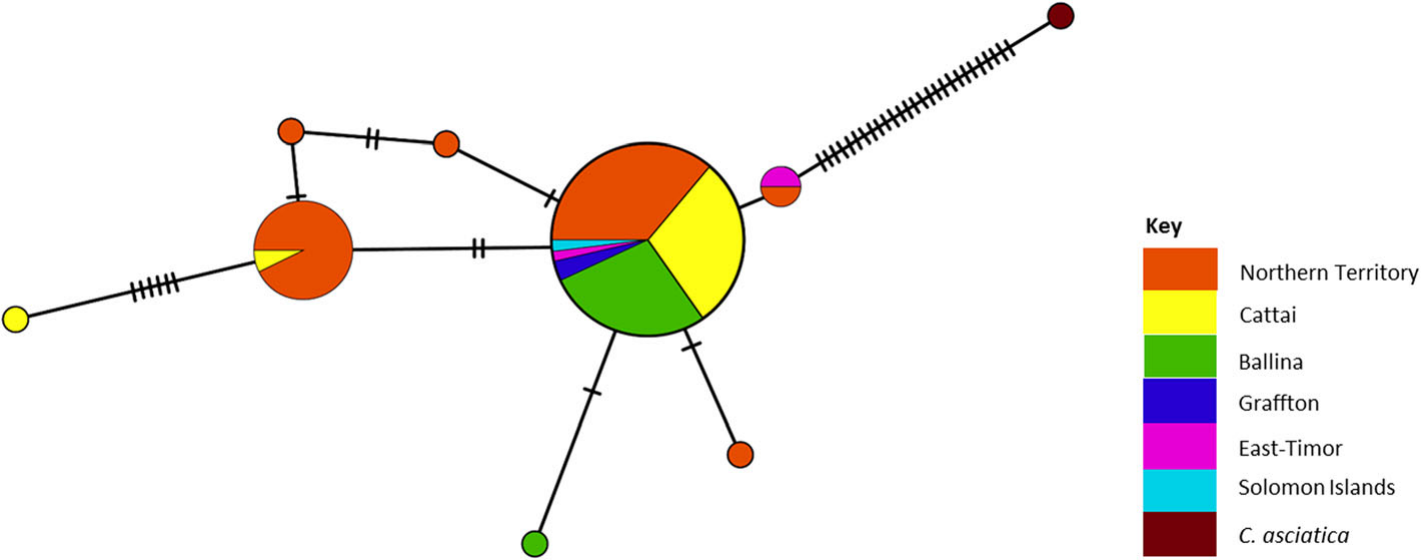
TCS network of the mitochondrial cytochrome oxidase I haplotypes from two biogeographic regions in Australia (NT and East Coast). The circles represent the different haplotypes. The size of each circle is proportional to the number of samples presenting the same haplotype. Each dash on a connecting line represents a mutation step leading to the differences between the sequences of haplotypes. Samples are coloured by region of collection. In brown (top right) is *C. asiatica*, the sister species to *C. brevitarsis*

A total of 9 haplotypes were identified (haplotype diversity Hd = 0.4478) (Fig. 4). All substitutions were synonymous and no stop codon was present. The first, most prevalent, haplotype (H1) consisted of 58 sequences mixed from the NT (*n* = 21), east coast (*n* = 35), East Timor (*n* = 1) and Solomon Island (*n* = 1) while the second most prevalent H2 consisted of 14 sequences almost exclusively from the NT (*n* = 13), and Cattai (*n* = 1). The rest of the haplotypes had 1 sequence each except H6 that had 2 sequences from East Timor and NT. H9 was an outgroup sequence from the closely related species *C. asiatica*.

To detect selection, tests of neutrality were estimated and gave significant negative values ((Tajima’s D = −2.66699) (Fu’s F test = −6.8522)] *P* <0.02).

## Discussion

The present study utilised GBS to investigate the phylogenetic relationships among *C. brevitarsis* populations across two biogeographic regions in Australia. The study aimed at providing clues to the geographic basis of the two episystems. Genotyping by sequencing, as a reduced representation library method [1], allows highly multiplexed sequencing of genomic subsets. The increase in sampling depth of the genomes (hundreds to thousands of loci) provides a powerful tool for studies of evolutionary, demographic and adaptive mechanisms at a population level [52]. Small sized insects like mosquitoes have been studied by reduced-representation library sequencing techniques such as RADSeq, either by pool [22] or individually [2, 3]. Despite their power, the application of NGS methods can be hampered by the limited amount of genomic DNA. Blair et al. [53] successfully isolated SNPs using RADseq after whole genome amplification of grey mouse lemur DNA with no significant genomic bias due to the previous genomic enrichment. The present study has isolated SNP markers using the GBS method from WGA DNA and used the resultant SNPs to genotype individual arthropods from two BTV episystems in Australia, in order to analyze their genetic structure. The approach did not require a previous reference genome.

Specimens from the same locations analyzed by microsatellite markers in a previous study revealed a panmictic population [15]. However, phylogenetic analysis conducted using both the isolated SNP markers and *mt*DNA in this study has revealed a sub population of *C. brevitarsis* in the NT. This sub-population was resolved as a separate cluster with the remainder of the NT samples gathering with samples from east coast. Using *mt*DNA (among other molecular markers) Tay et al. [54] demonstrated the existence of genetic discontinuity between the NT and eastern populations of *C. brevitarsis*. The discrepancy in terms of population structure between results obtained by microsatellite markers and SNPs and *mt*DNA could be expected. The mutation rates of SNPs (10 ^-8^–10^−9^) [55] and *mt*DNA (10^−8^) [56] are lower than that of microsatellites (range of 10^−3^–10^−5^) [28, 57]. These differences in mutation rates may reveal a difference in the targeted time scale. A higher mutation rate, as occurs for microsatellite markers, would detect more recent events than markers with slower mutation rates.

In this study, we focussed our sampling efforts on the two extreme biogeographic regions, NT and NSW of Australia, with limited sampling carried out in Qld. It is noticeable that, in the SNP analysis, all samples from Qld clustered with the NSW samples. The Gulf of Carpentaria separates Australia’s Top End, encompassing the northernmost section of the NT, from Cape York, a large peninsula at the far North of Queensland (Fig. 1). It is a biological barrier for many organisms (for a review, see [58]) including plants, birds, mammals [18] and insects [59]. For many organisms, the Cape York populations are closer to the Eastern region populations than to the northern Top End, with several examples of reduced gene flow between these populations [58]. We recommend further studies involving a wider sampling of *C. brevitarsis* from Queensland, including Cape York. This would shed light on the Carpentarian Gap as geographical barrier blocking gene flow between Qld and NT populations of *C. brevitarsis*.

The subpopulation of the NT could be indicative of founder effects. There is a possibility that some genes present in the founder population of the NT may not have been dispersed to the east coast population. Arrival of a unique population in the NT, possibly from Timor-Leste [15, 16] but not yet dispersed to eastern Australia, could also explain the existence of the separate NT sub-population. However, our sampling included only one site in the NT and one time point.

The F_ST_ estimate value obtained in this study suggests significant gene flow between the two populations. This shows that contact between the NT and the east coast populations still exists despite the presence of some alleles in the former population not assorted with the latter population. The presence of few loci showing strong evidence of selection could be due to the strong selection for local adaptation of alleles present in these loci. Further studies of the SNPs under selection are recommended. The negative Tajima’s D value could be indicative of either an expansion or a positive selection resulting from acquisition of a favourable trait.

The STRUCTURE results suggest that the individuals in this study had admixed genotypes and the individuals derived their ancestry from the two population clusters inferred in an unequal manner. The NT population seemed to derive its ancestry almost equally from both clusters while the east coast population seemed to derive its ancestry mainly from population cluster two. This could indicate that the NT is the ancestral population within Australia.

This study demonstrates that a combination of GBS to sample genomes densely [60], without need of reference genome, and the low bias of multiple displacement-based amplification [61] allow the use of small amounts of DNA (such as might be obtained from non-invasive sampling) for population genetics studies. The technical workflow described in this study is easily translatable to other species and will facilitate understanding of the distribution of pathogens spread by tiny vectors such as sandflies, fleas, lice, ticks, aphids, psyllids and mites. The method also has application to the study of any biological materials with limited amounts of genomic DNA.

## Conclusion

Although a heterogeneous population of *C. brevitarsis* (as shown in this study) could be a factor contributing to the presence of two separate BTV episystems in Australia (northern and eastern), these results should be taken with caution since individuals from the NT were sampled on only one occasion from a single site. More extensive sampling over a wider spatial and temporal range, including the northern part of Queensland (Cape York) would provide better resolution of the genetic connectivity of midge populations in Australia and shed light on the potential role of the Carpentarian Gap as barrier to gene flow. The epidemiological significance of the unique NT population, with loci under selection, also warrants investigation for vector competence studies comparing populations of *C. brevitarsis* from the NT and from eastern Australia. The described workflow is transferable for genotyping of small, non-model organisms, including arthropod vectors of pathogens of economic and medical importance.

## Additional files

Additional file 1: R script used to perform filtering of the isolated SNPs. This file has to be read with R. (R 14 kb) Additional file 2: The Matrix of data containing the list of SNPs selected after filtration crossed with selected samples. The ‘N’ tags the individuals where alleles are missing from the sequence reads. This could either be as a result of lack of a read at this site or a poor quality sequence, which cannot be called. (TXT 2744 kb) Additional file 3: Results of estimates of Hardy-Weinberg equilibrium tests conducted for each locus in the NT and East Coast populations (*P* < 0.01). Within each population, the monomorphic loci are not informative. (XLSX 299 kb) Additional file 4: BayeScan results for analysis for SNPs under natural selection. The first column is the tag of the selected reads. For each SNP, ‘prob’ represents the posterior probability of departure from neutrality; PO (posterior odds) is the ratio of posterior probabilities and is indicative of how more likely the model with selection is compared to the neutral model. The q value estimates the statistical significance of the deviation. The three lowest values are highlighted in yellow. The q value was used for ordering of the rows in the table. (XLSX 150 kb)

## Abbreviations

BTV: Bluetongue virus
GBS: Genotyping by sequencing
MDA: Multiple displacement amplification
NSW: New South Wales
NT: Northern territory
PCR: Polymerase chain reaction
Qld: Queensland
RADseq: Restriction-associated DNA sequencing
UNEAK: Universal network enabled analysis kit
WGA: Whole genome amplification
